# Pushing the envelope: Immune mechanism and application landscape of macrophage-activating lipopeptide-2

**DOI:** 10.3389/fimmu.2023.1113715

**Published:** 2023-01-24

**Authors:** Daoyong Liao, Xiaoling Su, Jingyun Wang, Jianwei Yu, Haodang Luo, Wei Tian, Zufeng Ye, Jun He

**Affiliations:** ^1^ The Affiliated Nanhua Hospital, Department of Clinical Laboratory, Hengyang Medical School, University of South China, Hengyang, China; ^2^ Department of Public Health Laboratory Sciences, School of Public Health, Hengyang Medical School, University of South China, Hengyang, Hunan, China; ^3^ Institute of Pathogenic Biology, Hengyang Medical School, Hunan Provincial Key Laboratory for Special Pathogens Prevention and Control, Hunan Province Cooperative Innovation Center for Molecular Target New Drug Study, University of South China, Hengyang, China

**Keywords:** macrophage-activating lipopeptide-2, *Mycoplasma fermentans*, inflammation, immunoreaction, application

## Abstract

*Mycoplasma fermentans* can cause respiratory diseases, arthritis, genitourinary tract infections, and chronic fatigue syndrome and have been linked to the development of the human immunodeficiency virus. Because mycoplasma lacks a cell wall, its outer membrane lipoproteins are one of the main factors that induce inflammation in the organism and contribute to disease development. Macrophage-activating lipopeptide-2 (MALP-2) modulates the inflammatory response of monocytes/macrophages in a bidirectional fashion, indirectly enhances the cytotoxicity of NK cells, promotes oxidative bursts in neutrophils, upregulates surface markers on lymphocytes, enhances antigen presentation on dendritic cells and induces immune inflammatory responses in sebocytes and mesenchymal cells. MALP-2 is a promising vaccine adjuvant for this application. It also promotes vascular healing and regeneration, accelerates wound and bone healing, suppresses tumors and metastasis, and reduces lung infections and inflammation. MALP-2 has a simple structure, is easy to synthesize, and has promising prospects for clinical application. Therefore, this paper reviews the mechanisms of MALP-2 activation in immune cells, focusing on the application of MALP-2 in animals/humans to provide a basis for the study of pathogenesis in *Mycoplasma fermentans* and the translation of MALP-2 into clinical applications.

## 1 Introduction

Mycoplasma is the smallest prokaryotic cellular microorganism with the smallest available genome, capable of growing and reproducing in cell-free conditions. Mycoplasmas, diverse and widespread, pose a significant risk to humans and livestock. More than 20 species of mycoplasma have been isolated from humans, mainly colonizing the oropharynx, upper respiratory tract, and genitourinary tract (1). *Mycoplasma fermentans (M. fermentans)* often resides in the mucous membrane of healthy people and patients with congenital immunodeficiency, causing respiratory diseases, arthritis, genitourinary tract infections, chronic fatigue syndrome, and is associated with *human immunodeficiency virus* (HIV) infection and development (2–4).

Because mycoplasma lacks a cell wall, its outer membrane lipoproteins are believed to be one of the main factors in inducing inflammation in the organism and contributing to disease development. Therefore, studying lipoproteins in the outer membrane is beneficial for better understanding mycoplasma’s pathogenic mechanisms. Macrophage-activating lipopeptide-2 (MALP-2) derived from the outer membrane of *M. fermentans* with host immune modulating function, named initially “MDHM” meaning “Mycoplasma-derived high molecular weight material” (5). Mühlradt PF et al. named it MALP-2, MALP for macrophage activating lipopeptide, and 2 indicates 2 kDa. MALP-2 comprises a 13-residue peptide GNNDESNISFKEK attached to a (2R)-3-((2-amino-3-oxobutyl)thio)propane-1,2-diyl dipalmitate lipid chain, N-terminal fatty acid chain containing two diacylglycerol-cysteine structures anchored to the cell membrane (6, 7). The N-terminal amino acid sequence of MALP-2 is identical to that of the *M. fermentans* proteins M161Ag and P48, suggesting the lipopeptide is likely derived from either M161Ag or P48. Studying these precursor lipoproteins will facilitate a better understanding of the function of MALP-2 (8).

Mühlradt PF et al. compared synthetic dipalmitoyl MALP-2 with Mycoplasma-derived MALP-2 using a bioassay and found that both lipopeptides had the same dose dependence (6). Both had half-maximal responses at 10^-11^ M concentrations, suggesting the biological activities of both are approximately the same, providing experimental evidence for the subsequent application of synthetic MALP-2 (6). The lipid portion of MALP-2 has an asymmetric C atom. Takeuchi O et al. synthesized two stereoisomers of MALP-2 with different lipid fractional conformations: R-MALP and S-MALP, and found that both were able to activate macrophages for biological effects, and the induction effect of R-MALP was much higher than that of S-MALP (9). Synthetic MALP-2 also has an endotoxin-like activity similar to LPS and induces a local cutaneous Shwartzman reaction in rabbit and murine lethal shock, but the response is weaker than LPS. Interestingly, when MALP-2 and LPS were used in low concentrations to stimulate spleen lymphocytes in mice, MALP-2 had a superior ability to activate the cells than LPS (10). MALP-2 is effective at very low concentrations (ng/ml, 0.5 µg per mouse). Also, MALP-2 is extremely lipophilic and should be handled carefully and placed in glass vials. Since it is active at very low concentrations, i.e., at high dilutions, these low amounts of material tend to become adsorbed to pipette tips and plastic or glass containers. To avoid this, prepare several dilution steps of the MALP-2 stock solution with a medium containing 5% autologous serum or buffers with 2% human serum albumin. The similarities and differences between MALP-2 and LPS deserve further study and comparison (10).

MALP-2 activates a variety of immune cells in humans, such as indirectly enhancing cytotoxicity of NK cells, promoting phagocytosis and oxidative burst of neutrophils (PMN), upregulating surface markers on lymphocytes, enhancing antigen presentation on dendritic cells, and inducing immune inflammatory responses in sebaceous gland cells and mesenchymal cells, the most important of which is the bidirectional regulation of inflammatory reactions in monocytes/macrophages. Considering the immune cell activation properties of MALP-2 and the induced immune response, it is likely that this lipopeptide is critical for the interaction between mycoplasma and immune cells. In addition, the immune activity of MALP-2 has led to promising discoveries in applications such as vaccine adjuvant, vascular regeneration and healing, wound and bone healing, and infection prevention. In conclusion, this review focus on the application of MALP-2 and the mechanism to activate immune cells.

## 2 Mechanism of MALP-2 activation of immune cells

### 2.1 MALP-2 regulates inflammatory responses in monocytes/macrophages

**Figure 1:**
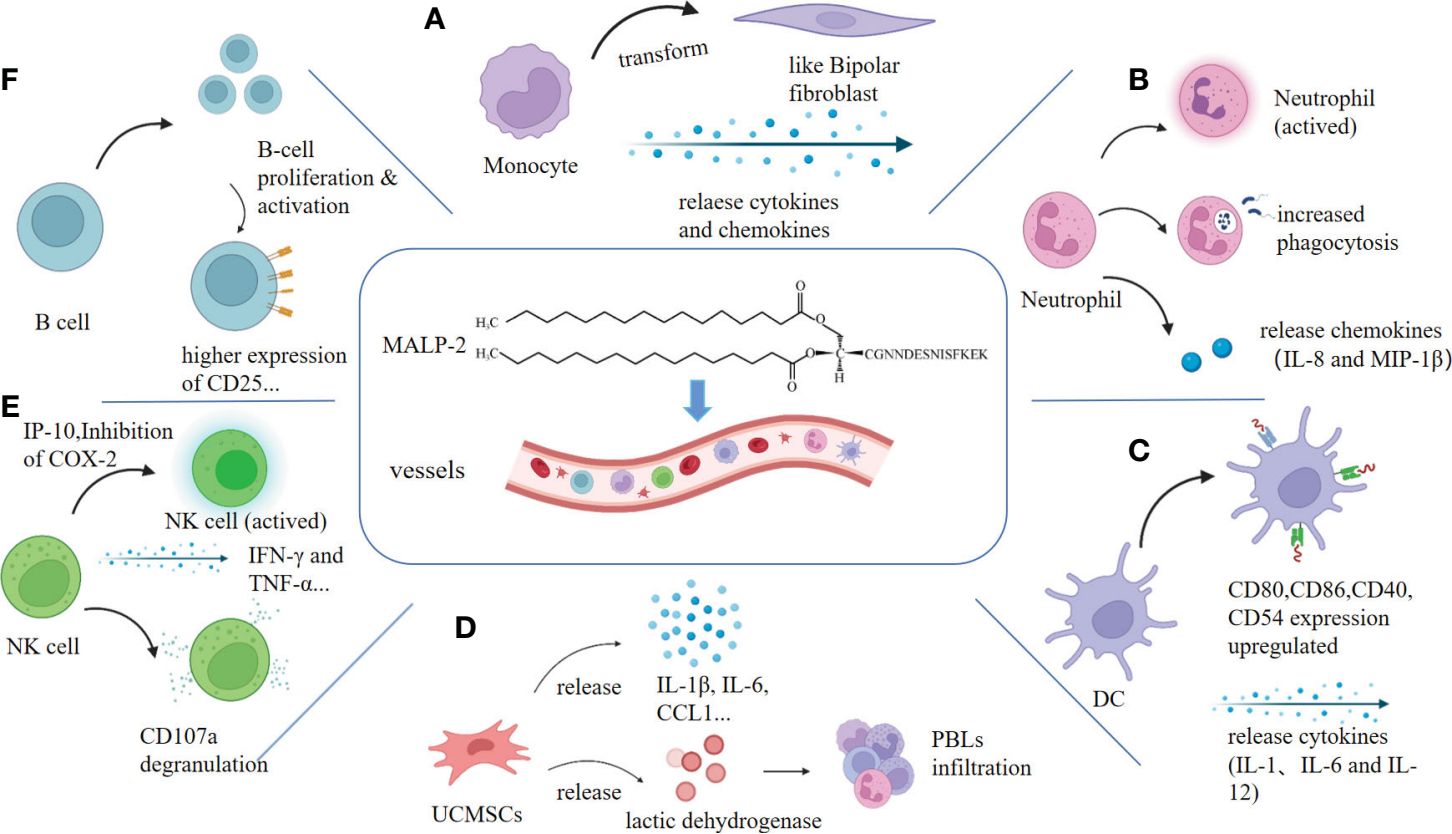
MALP-2 activates immune cells. Clockwise: **(A)** MALP-2 activated monocytes release cytokines and chemokines, causing monocytes to exhibit a bipolar fibroblast-like morphology. **(B)** MALP-2 enhanced the phagocytosis of PMN and activated PMN to secrete IL-8 and MIP-1β. **(C)** MALP-2 induced dendritic cells to release cytokines and stimulated upregulation of expression of co-stimulatory molecules and others. **(D)** MALP-2 activated NK cells to release IFN-γ and TNF-α in the presence of COX-2 inhibitors, enhancing CD107a degranulation from NK cells. **(E)** MALP-2 induced the release of cytokines and chemokines and promotes lactate dehydrogenase release from damaged UCMSCs. **(F)** MALP-2 activated B cells and increases the expression of their surface activation markers, such as CD25, which significantly enhances B cell proliferation.

MALP-2 is one of the classical inducers that induce monocytes/macrophages to release cytokine (11). After co-culture with MALP-2, monocytes showed enhanced adhesion and bipolar fibroblast-like morphology. MALP-2 markedly induced the expression of pro-inflammatory factors TNF-α, IL-6, IL-1β, COX-2, and chemokines CXC, MCP-1, MIP-1α, MIP-1β in monocytes in a dose-dependent manner (12) (**Figure 1A**). Heme oxygenase-1 (HO-1) expression in monocytes is known to mediate anti-inflammatory effects. Studies have found that MALP-2 activates PI3K *via* TLR2/6, Btk, and Malandc-Src, which initiates the activation of basic leucine zipper transcription factor NF-E2-related factor 2 (Nrf2), thereby MALP-2 inducing monocyte express HO-1 and increasing the level of HO-1 enzymatic activity (13, 14). The association of Btk, c-Src, and Mal is required to activate PI3K *via* the MALP-2-triggered signaling pathway, Btk, c-Src, Mal, and MyD88 form a physical complex, which further induces Nrf2 translocation to the nucleus and binding to the ARE-site to stimulate HO-1 expression (15). HO-1 prevents the overactivation of monocytes/macrophages and forms a feedback loop to negatively regulate the host inflammatory response, thereby avoiding potentially harmful immune outcomes (16). During mycoplasma infection, matrix metalloproteinases (MMPs) participate in various biological effects, such as degrading the extracellular matrix engaged in inflammatory response and angiogenesis. And studies have found that MALP-2 induces the expression of MMP-9 in THP-1 cells *via* the MAPKs pathway (17, 18). In summary, MALP-2 plays a dual role in modulating the inflammatory response of the organism.

**Figure 2:**
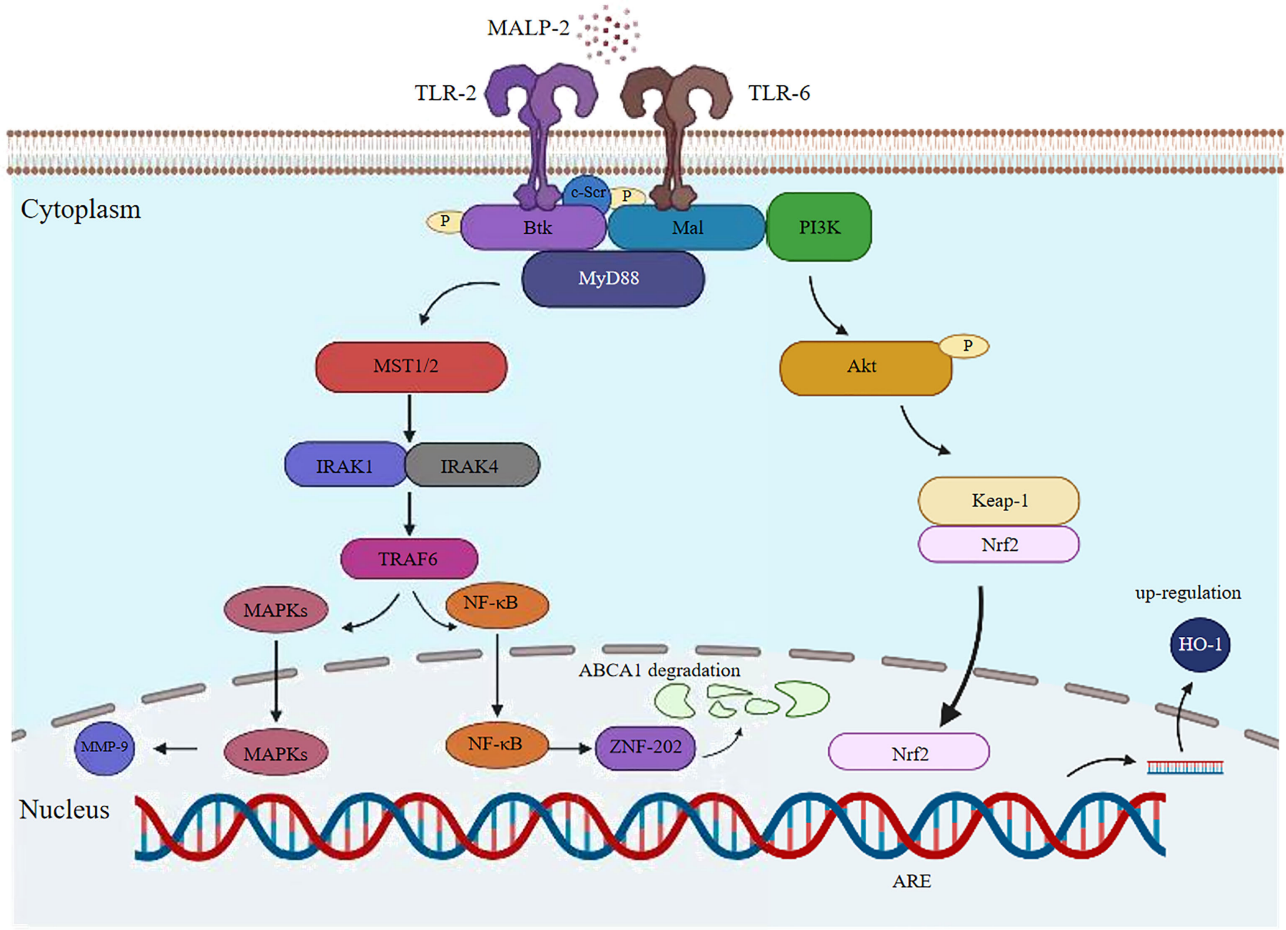
Potential signaling pathway induced by MALP-2 in THP1 cells. MALP-2 induces MMP-9 expression in THP-1 cells *via* the MAPKs pathway; MALP-2 can decrease ABCA1 expression and subsequent cholesterol efflux through activation of the TLR2/NF-κB/ZNF202 signaling pathway in THP-1 macrophages; The cytoplasmic adaptor Mal recruits the TLR2/6 heterodimer in response to MALP-2 stimulation. c-Src, Btk, Mal and MyD88 form a physical complex to activate PI3K, which further induces Nrf2 translocation to the nucleus and binds to the ARE-site to stimulate HO-1 expression.

MALP-2 induced the activation of MAPK family members extracellular signal regulated kinases 1 and 2, c-Jun NH2-terminal kinase, and p38 and induced NF-κB and AP-1 transactivation in macrophages. MAPKs are essential in the recruitment and activation of monocytes/macrophages in infected tissues and in the induction of pro-inflammatory cytokine and chemokine expression by macrophages, which provides for leukocyte infiltration and inflammatory responses after mycoplasma infection (19, 20). ATP-binding cassette transporter A1 (ABCA1) is a transmembrane protein that plays a pivotal role in preventing foam cell formation and promoting reverse cholesterol transport (RCT) (21). MALP-2 reduces ABCA1 expression and inhibits subsequent cholesterol efflux in a concentration-dependent and time-dependent manner by activating the TLR2/NF-κB/ZNF202 signaling pathway in THP-1 macrophages (22). The signaling pathway of MALP-2 activation is summarized in (**Figure 2**).

### 2.2 MALP-2 promotes phagocytosis and oxidative burst of PMN

10 ng/mL MALP-2 induces neutrophil (PMN) morphology elongation and secretion of IL-8 and MIP-1β; IL-8 acts as an amplification loop in acute phase neutrophil recruitment (23, 24). The antimicrobial function of PMN is mainly the phagocytosis of invading pathogens. After co-incubating PMN for 75 min, MALP-2 significantly enhanced the phagocytosis of staphylococcus aureus in PMN. Expression of CD11b upregulated, and CD62L downregulated on PMN (**Figure 1B**). MALP-2 suppressed structural apoptosis in PMN. However, MALP-2 had a short-term inhibitory effect on resting PMN apoptosis but a more durable protective effect on spontaneous PMN apoptosis after endothelial migration (24).

Oxidative burst is necessary for PMN kills phagocytosed microorganisms. MALP-2 can promote fMLP to cause an enhanced oxidative burst in PMN and generate reactive oxygen species to destroy melanoma cells. fMLP is a potent neutrophil chemotactic peptide that can induce leukocyte chemotaxis and activate macrophage (25, 26). Compared to MALP-2 (10 ng/ml), the TLR1/2 agonist triacylated bacterial lipopeptide Pam3CysSK4 (Pam3) requires higher concentrations (10 μg/mL or more) to activate PMN, and MALP-2 - unlike the synthetic TLR1/2 ligands Pam3 - occurs naturally (24, 27). As a result, MALP-2 has an advantage over other TLR agonists in terms of the quantity used, and the enhanced phagocytosis of PMN induced by MALP-2 provides a basis for subsequent application in anti-infection.

### 2.3 MALP-2 enhances the antigen presentation in dendritic cells

Dendritic cells (DCs) are the most potent antigen-presenting cells (APCs), stimulating primary T cells and thus inducing immune responses against microbial and tumor antigens (28). *In vitro* experiments, MALP-2 stimulated the expression of MHC class II molecules, co-stimulatory molecules, and adhesion molecules in mouse DC. MALP-2 enhanced the secretion of cytokines IL-1, IL-6, and IL-12 and increased the stimulatory activity of DCs on naive and antigen-specific T cells (**Figure 1C**). MALP-2 also leads to the transition of DCs from the proteasome to the immunoproteasome through the up-regulation of LMP2, LMP7, and MECL1 (29). The maturation of DC cells stimulated by MALP-2 is closely related to the subsequent use of MALP-2 in adjuvant applications, where more mature DC cells improve antigen presentation and vaccine efficacy.

### 2.4 MALP-2 indirectly enhances the cytotoxicity of NK cells

The TLR-2 receptors are present on the surface of NK cells. Thus MALP-2, an agonist of the TLR-2/6 receptors, is believed to activate NK cells. However, Sawahata R et al. found that MALP-2 could not induce cytotoxic responses in murine-derived NK cells *in vitro*, which is similar to Müller C’s research that MALP-2 could not directly activate NK cells (11, 30). Nevertheless, in the mouse model of pancreatic cancer, MALP-2 inhibited the growth and development of pancreatic cancer, most likely by stimulating the activity of NK cells and T cells (31). In phase I/II clinical trials in patients with incompletely resectable pancreatic cancer, postoperative NK cell activity was substantially higher in patients treated with MALP-2 than in control subjects (32). In a rat metastasis model, MALP-2 treatment caused a considerable increase in the accumulation of monocytes and NK cells at the metastatic site (33). These studies suggest that MALP-2 affects tumor suppression of NK cells. It may be attributed to other factors that prevent MALP-2 from directly activating NK cells *in vitro*, thus presenting completely different experimental results *in vivo* and *in vitro*.

MALP-2 invoked the release of various cytokines and chemokines from monocytes, including IL-10, MIP-1α, and COX-2, of which both IL-10 and MIP-1α greatly enhanced the cytotoxicity of NK cells; in contrast, the expression of COX-2 in monocytes led to the production of PGE2, a potent inhibitor of NK cell activity (13, 34, 35). Therefore Müller C et al. cultured monocytes and NK cells in COX-2 inhibitors and found that MALP-2 induced chemotaxis of NK cells (11). Moreover, MALP-2 enhanced NK cell cytotoxicity, upregulated NK cell surface CD69 expression, and enhanced IP-10-mediated degranulation on NK cells by CD107a, a sensitive marker of NK cell degranulation that reflects NK cell toxicity and cell killing activity. NK cells also produce essential cytokines for tumor surveillance, such as IFN-γ and TNF-α (11, 36) (**Figure 1E**). MALP-2 can indirectly activate NK cells by stimulating monocytes and enhancing the cytotoxicity of NK cells to enable them to play an influential role in immune killing.

### 2.5 MALP-2 upregulates surface markers on B lymphocytes

Studies in T-cell and B-cell deficient mice confirmed that follicular B cells were targeted cell subsets by MALP-2, and B-1A and marginal zone B cells also respond to MALP-2. B cell proliferation was significantly enhanced by MALP-2 at 100 or 1000 ng/mL, with 45% more enlarged B cells and increased intracellular granulation. The B cell subsets also showed higher expression levels of CD25, CD19, MHC I, MHC II, CD80, CD86, and CD40, and B cells were activated directly by TLR2 without T cell assistance (37) (**Figure 1F**). High expression of CD25 indicates B-cell activation, and upregulation of CD19 expression correlates with a lower threshold for antigen receptor stimulation, suggesting that MALP-2 enhances B-cell sensitivity to antigens (38, 39). MALP-2-mediated activation of B cells through TLR2/6 is critical for adjuvanticity. B cell stimulation by pattern-recognition receptors seems to be a fundamental mechanism that MALP-2 can exploit to improve the immunogenicity of vaccine formulations.

### 2.6 Immunological effects of MALP-2 on other cells

MALP-2 induces different immune effects in other human cells. MALP-2 activates inflammatory signals in human sebaceous cells by upregulating stearoyl-CoA desaturase (SCD) and fatty acid desaturase 2 (FADS-2), and both enzymes are only associated with lipid unsaturation, confirming the sequential induction of SCD and FADS2 by MALP-2 (40). Propionibacterium acnes induces an inflammatory response around the sebaceous glands leading to acne worsening mainly through the secretion of pro-inflammatory lipids and various cytokines, whereas MALP-2 upregulates sebocyte cells TLR-2 mRNA levels, stimulating the release of cytokines IL-6 and IL-8, thereby exacerbating the inflammatory response and acne worsening (41).

MALP-2 activates the NF-κB pathway to induce secretion of IL-6, IL-8, and Granulocyte-macrophage Colony Stimulating Factor (GM-CSF) in amniotic mesenchymal cells, thereby causing a robust inflammatory response leading to premature rupture of the fetal membranes (42). MALP-2 actives umbilical cord mesenchymal stem cells (UCMSCs) to express pro-inflammatory cytokines, including IL-1β, IL-6, IL-8, IL-10, and two chemokines, CCL1 and CCL4. And it can increase the proliferation of peripheral blood leukocytes (PBLs) and promote the release of lactate dehydrogenase from damaged UCMSCs (**Figure 1D**). In addition, it promotes immune status without affecting the differentiation capacity of UCMSCs (43). The action on mesenchymal cells facilitates the use of MALP-2 in cell therapy.

## 3 Immunological application of MALP-2

### 3.1 MALP-2 inhibits tumor growth and metastasis

Intratracheal application of MALP-2 in mice resulted in a significant influx of neutrophil- and macrophage-dominated leukocytes into the interstitial lung, both of which are essential for anti-tumor defense (44). Intraperitoneal administration of MALP-2 in mice treated with *in situ* pancreatic cancer revealed a statistically significant reduction in tumor growth and extended survival time in mice. Flow cytometry analysis showed a notable increase in CD8^+^ lymphocytes, NK cells, and T helper cells. Immunohistochemical results showed that the mice exhibited progressive, fused tumor cell necrosis with prominent lymphocyte infiltration (31). The phase I/II trial of intra-pancreatic cancer patients treated with MALP-2 also found no toxicity observed in patients treated with up to 20 mg of MALP-2. The high mean survival of 17.1 months demonstrated that MALP-2 extends the patient’s life expectancy. However, the paper’s cohort was too small to conclude (32). In addition to tumor suppression, MALP-2 also resists tumor metastasis to the lungs. The mouse experiments confirmed that MALP-2 administered 1 or 3 days after MADB106 tumor cell injection did not affect lung metastasis of tumor cells, but MALP-2 injected 60 minutes earlier or simultaneously with MADB106 tumor cells increased the number of monocytes and NK cells in lung tissue. Eventually, lung metastasis of cancer cells decreased, these suggest that MALP-2 has anti-tumor metastatic effects (33). Therefore, MALP-2 has the potential to be used as a novel agent for early anti-tumor, tumor inhibition, and prevention of tumor metastasis.

**Figure 3:**
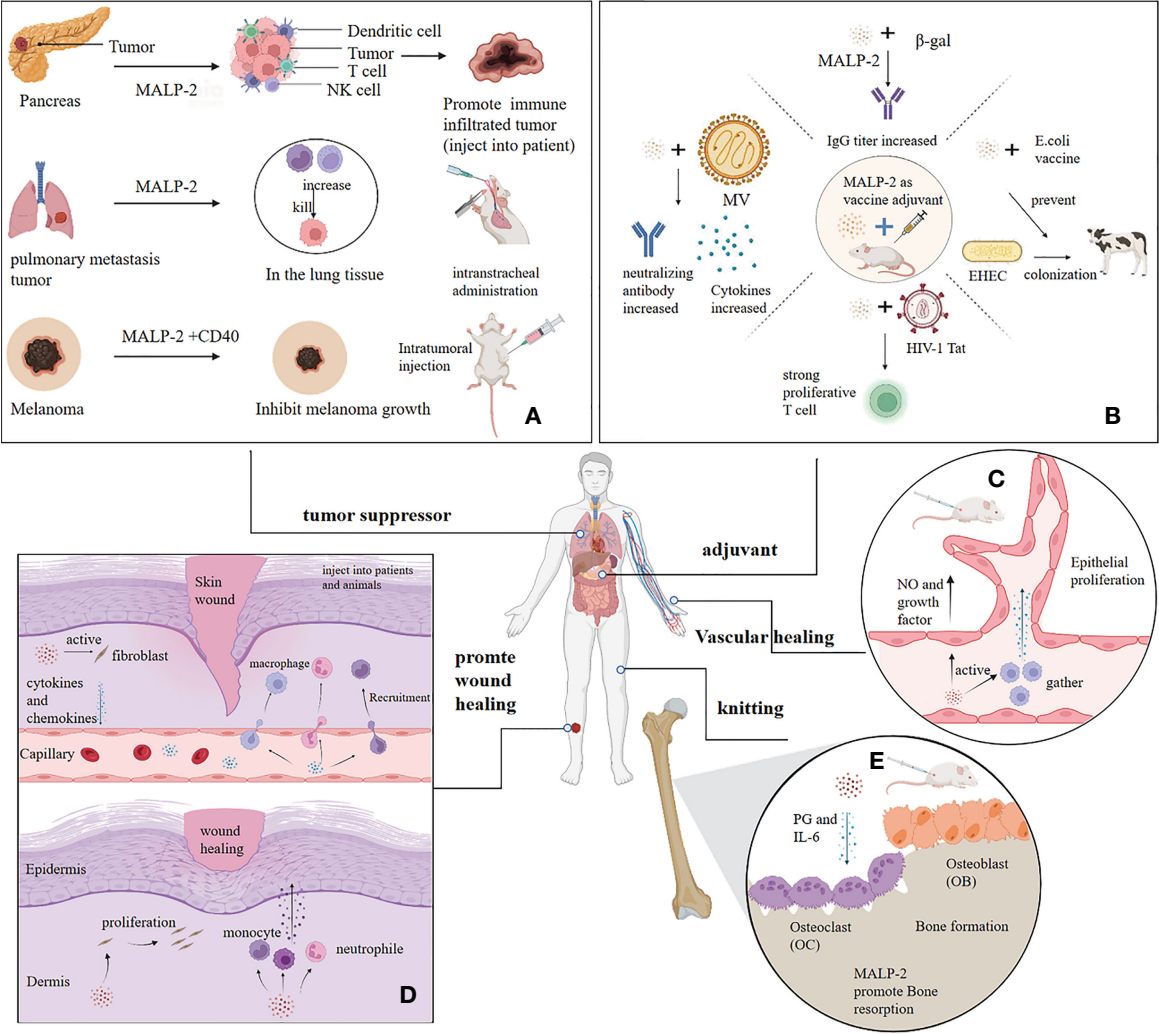
Application of MALP-2. **(A)** MALP-2 inhibited the growth of pancreatic cancer tissue in patients and prevented lung metastases in mice, and MALP-2, combined with CD40, significantly suppressed melanoma growth in mice. **(B)** MALP-2 can enhance the immune response and act as an adjuvant when used with various vaccines. **(C)** MALP-2 recruited macrophages to release cytokines to induce vascular endothelial cell proliferation and promote angiogenesis; MALP-2 stimulated endothelial cells to release NO and cytokines. **(D)** MALP-2 stimulates skin fibroblasts to release cytokines and chemokines, thereby recruiting granulocytes, monocytes, and macrophages to the wound; MALP-2 induces fibroblasts to proliferate and stimulates recruited immune cells to release growth factors for wound healing. **(E)** MALP-2 increases bone resorption activity of osteoclasts by inducing the release of PGE and IL-6, which in turn accelerates bone healing in mouses.

MALP-2 enhances the oxidative bursts of PMN to produce reactive oxygen species for destroying melanoma cells (25, 26). Schill T et al. raised theoretical questions about the study of MALP-2 inhibition in melanoma because the TLR2/6 pathway is expressed not only in immune cells but also in some tumor cells, including B16-F10 melanoma (45). Activation of TLR in tumor cells induces MAPK signaling and NF-κB activation, which may promote cancer progression. TLR agonists act as “danger signals” to convert the tolerogenic microenvironment of tumors into an immunogenic one. In some cases, does the stimulation of TLR by MALP-2 act as a tumor growth-enhancing adjuvant rather than a specific inducer of anti-tumor activity (45)? The MTT proliferation assay *in vitro* found no detectable effect of MALP-2 on melanoma cell proliferation and no direct promotion of melanoma metastasis, which requires further validation *in vivo*. The histological analysis detected no particularly significant increase in inflammatory cells near the tumor after MALP-2 treatment, suggesting that the immune response may not specifically target tumor cells (46). Similarly, a study found no melanoma inhibition was observed with five repeated intratumoral injections of MALP-2. However, the combination of CD40 and TLR agonists markedly suppressed melanoma growth in mice (47) (**Figure 3A**). In conclusion, both the timing of MALP-2 administration and the co-administered tumor suppressors impact the anti-tumor capacity of MALP-2. TLR2/6 stimulation by MALP-2 exerts complex and pleiotropic effects on several cell types, which may even antagonize each other, ultimately making it possible for MALP-2 to exhibit a dual effect on tumors *in vivo*.

MALP-2 indirectly enhances the cytotoxicity of NK cells in the presence of COX-2 inhibitors, so blocking COX-2 administration is considered a promising approach for MALP-2 adjuvant oncology therapy. Actinic keratosis, a precancerous lesion caused by UV light, has been successfully treated with the COX-2 inhibitor diclofenac (48). The above results provide a new research direction to consider the co-use of MALP-2 with tumor suppressors in future cancer immunotherapy regimens for better therapeutic effects.

MALP-2 shows Antitumor activity by inducing cytokine secretion from specific human immune cells (for example, interferon-a, interleukin IL-12, TNF-a). When MALP-2 is used with chemotherapy drugs, the response to chemotherapy produces massive release and processing of tumor antigens. This response leads to a state in which the immune system is primed (via *in situ* vaccination) to respond to exogenous macrophage activation signals with potent specific antitumor effects (49).

### 3.2 MALP-2 improves infection and inflammation in the lungs

Intratracheal MALP-2 application enhanced the recruitment of leukocytes such as PMN, macrophages, and lymphocytes into the alveolar space of mice without detectable systemic side effects (44). One study found that pulmonary immunostimulation with MALP-2 before infection with *Streptococcus pneumoniae* improved local host defenses and increased survival in murine pneumococcal pneumonia. MALP-2 reduced the number of lung bacteria, altered the bacterial distribution in the lung, and decreased the bacterial blood load 72 hours after infection. MALP-2 increased the release of CCL5 and decreased IL-10 in the lung tissue (50). Reduced IL-10 was accompanied by reduced pulmonary bacterial counts and lethality in murine pneumococcal pneumonia, while increased CCL5 blocked the transition to lethal Streptococcus pneumoniae pneumonia (51, 52). MALP-2 also increased the expression of mRNA and protein of TLR-2 in alveolar epithelial cells, improving the first line of host defense against pathogen recognition (50).

Local immuno-suppression caused by the pulmonary influenza virus has been identified as a major cause of secondary bacterial pulmonary infections. It is often associated with high morbidity and mortality when two overlapping infections occur (53). MALP-2 has also been shown to attenuate secondary infection of Streptococcus pneumoniae. Local immunostimulation of MALP-2 attenuated hypothermia and weight loss in mice, resulting in increased survival in mice with dual infection. Specifically, MALP-2 notably reduced the number of bacteria in lung tissue and increased PMN to the alveolar space, with no detectable side effects in the experiment (54). Thus, exploring the use of MALP-2 prophylactic immunization of the lungs may improve the prognosis of pneumonia in high-risk patients and offer the prospect of clinical application for the prevention of secondary bacterial lung infections in patients with a pandemic or seasonal influenza. *Pseudomonas aeruginosa* (*P. aeruginosa*) is a leading pathogen of hospital-acquired infections and endangers immunocompromised and critically ill patients. Treatment of rats infected with *P. aeruginosa* using MALP-2 at different time points resulted in a significant reduction of P. aeruginosa CFU and lung tissue inflammation in the lungs of rats, and rats were noticeable relief of infection symptoms such as weight loss (55). MALP-2 suppressed the growth of Mycobacterium bovis BCG in alveolar macrophages, and MALP-2 treatment reduced macrophage bacterial load on days 3 and 7 after infection while enhancing the release of TNF-α, IL-6, and IL-10. The results suggested that MALP-2 can effectively reduce bacterial load and activity in pulmonary macrophages and may apply to developing new anti-tuberculosis drugs by modulating the anti-mycobacterial defense mechanism of macrophages (56–58). In summary, MALP-2 upregulates and activates the expression of TLR2/6, thereby enhancing the host’s immune response to foreign pathogens, the host’s defense against and clearance of multiple pathogens in the lung, and ultimately shortening the course of the host’s disease and successfully reducing morbidity and mortality in experimental mice.

In addition to preventing and mitigating pulmonary infections, MALP-2 promoted recovery and improved survival in mice with systemic inflammation. In the later stages of trauma, the immune response is dysfunctional, predisposing to infectious complications such as sepsis and organ dysfunction. The lung becomes a major target organ for inflammation-related tissue damage because of the high exposure of inflammatory mediators and immune cells to the bloodstream and through the lungs (59, 60). Intratracheal injection of MALP-2 into mice induced the synthesis of several cytokines and chemokines, resulting in a massive influx of leukocytes into the interstitial lung, interestingly MALP-2 reduced pulmonary infiltration of PMN in the systemic inflammatory response (44). Animal experiments revealed an increased survival in MALP-2-treated septic mice with both cecum ligation and puncture compared to control, accompanied by reduced plasma MCP-1 levels, decreased chemokine and cytokine release from alveolar and peritoneal macrophages, and diminished pulmonary infiltration of PMN. These results demonstrate that MALP-2 attenuates the pulmonary inflammatory response in septic mice. The decrease in neutrophil infiltration in lung tissue is mainly induced by the action of MALP-2 on peritoneal macrophages. The decrease in systemic immune response due to systemic inflammation also indirectly led to a decrease in pulmonary neutrophil infiltration (61). MALP-2 also reduces mortality and lung and liver injury in mice with trauma and sepsis. MALP-2 should be used at the earliest sepsis diagnosis, and a larger cohort is needed to determine the optimal dose of MALP-2 (62, 63). Intra-pulmonary neutropenia is inevitably strongly associated with increased survival in septic mice. More studies are needed to confirm the mechanism of action in MALP-2, specifically on how to fight infection, anti-inflammation, and reduce mortality in model animals.

MALP-2 also plays a role in aseptic inflammation. MALP-2 attenuates inflammatory responses, alleviates symptoms in a mouse model of allergic asthma, and markedly stimulates IL-10 secretion by mast cells in mice (64). Pfeifer R et al. found that MALP-2 pretreatment attenuated liver, lung, and systemic inflammatory responses in a mouse model of hemorrhagic shock (HS). These protective mechanisms were associated with a decrease in the pro-inflammatory cytokine IL-6. Moreover, MPO activity was elevated dramatically in the liver of mice treated with MALP-2, suggesting that MALP-2 affects sterile inflammatory pathways (65). MALP-2, as a highly active substance, maybe a new option for treating sterile inflammation after HS or severe injury.

### 3.3 MALP-2 is a promising vaccine adjuvant

MALP-2 is an adjuvant that enhances mucosal, humoral, and cellular immune responses. MALP-2, which is aliphatic, is readily absorbed across cellular and basement membranes and acts as a mucosal immune adjuvant (66). MALP-2 combined with β-Galactosidase (β-gal) stimulated effective IgA production in distant mucosa of mice such as vaginal lavage after intranasal injection, and the combination of MALP-2 with Cholera Toxin Subunit B (CTB) also stimulated effective IgA production in the mucosa (67).

MALP-2 was combined with CTB to treat cells from the spleen and lymph nodes of mice. Levels of IFN-γ, IL-2, IL-4, and IL-10 are measured in the cell supernatants, with IL-10 being the most significantly elevated cytokine, indicating that immunized mice produced a mixed Th1/Th2 response, with a predominantly Th2-type reply because the cytokines secreted by Th1 are mainly IFN-γ, in contrast, those secreted by Th2 include IL-4, IL-5, IL-10, and IL-13 (67, 68). Co-inoculation of MALP-2 with the HIV-1 Tat protein stimulated a strong Th1-dominated Tat-specific proliferative T cell response. Therefore, MALP-2, with Tat protein, may be a promising vaccine candidate for preventing and treating AIDS (69, 70).

The inoculation of MALP-2 with β-gal intranasally induced an effective proliferative response in lymph node cells and splenocytes in a dose-dependent manner, with an approximately 100-fold increase in β-gal specific IgG titers in lung lavage fluid (67). Co-inoculation of MALP-2 and Measles Virus (MV) enhanced the neutralizing antibody response in mice. Co-inoculation of MALP-2 induces protective immunity in the absence but not in the presence of MV specific antibodies (71). For Enterohemorrhagic Escherichia coli (EHEC), MALP-2 effectively induces an immune response against the C280 endosomal protein and E. coli secretory protein B of EHEC and increases the production of antigen-specific serum antibodies. MALP-2 likely helps prevent outbreaks of EHEC infections in humans and is used in animal husbandry to protect cattle from colonization by EHEC O157 (72). No adverse reactions or acute or chronic toxicity were observed during the animal injection, probably due to the inherent weak immunogenicity of MALP-2 and the short peptide that minimizes specific immune responses, which gives MALP-2 an advantage over conventional protein adjuvants (67, 69). Overall, MALP-2 is a novel and effective vaccine adjuvant that can be used to design novel vaccination strategies (**Figure 3B**).

### 3.4 MALP-2 promotes the regeneration and healing of blood vessels

Reendothelialization is clinically essential in promoting vascular healing. Restenosis and neointima formation due to the proliferation of smooth muscle cells are clinical problems. Nowadays, drugs inhibit neointima formation while hindering reendothelialization, whereas MALP-2 promotes reendothelialization after vascular injury and simultaneously inhibits neointima formation (73). Activation of the TLR2/6 receptor directly promotes angiogenesis due to the driving effect of GM-CSF, which recruits immune cells for pathogen defense and tissue regeneration (74). Troidl K et al. investigated the impacts of MALP-2 on collateral arteries and blood flow restoration in Hypercholesterolemic apolipoprotein E (ApoE)-deficient mice with ligated femoral arteries. The research found that MALP-2 remarkably promoted the proliferation of endothelial cells in the intima and the aggregation of macrophages around the collateral arteries. MALP-2 also induced the growth of preexisting collateral arteries in the upper hind limb and increased capillary density in the lower hind limb, ultimately improving blood flow recovery in mice (75). For endothelial cells, MALP-2 increases nitric oxide synthase phosphorylation and NO release, thereby improving experimental vasodilation in isolated mesenteric arteries. MALP-2 may contribute to blood flow restoration by enhancing collateral artery growth, which is beneficial for therapeutic angiogenesis, alleviating tissue ischemia, and improving perfusion recovery during acute events such as myocardial infarction and stroke (75). (**Figure 3C**)

Given the ability of MALP-2 to promote vascular healing, Laschke MW et al. applied different doses of MALP-2 to mice implanted with porous polyethylene and found that MALP-2 dose-dependently promoted angiogenesis at the implant site. There was a significant increase in the density of functional microvessels at the border and center of the implant. The implants were surrounded by granulation tissue, which exhibited a significantly higher density of CD31-positive microvessels and the number of F4/80-positive macrophages compared to controls (76). MALP-2 effectively stimulates early angiogenesis at the implant site in mice without causing local or systemic side effects. Its immunostimulation also facilitates host defense and bacterial clearance at the implant site (76). Grässer C et al. used MALP-2 when implanting polyurethane scaffolds with microvascular fragments into mice’s dorsal skin fold chambers. This study found that MALP-2 promoted apoptosis of endothelial and perivascular cells in microvascular fragments and also impaired the ability of adipose tissue-derived microvascular fragments to develop a new microvascular network within the implant (77). Despite MALP-2’s pro-angiogenic properties, implant functional microvessel density was reduced, which is inconsistent with Laschke’s findings (76, 77). They suggest that the apoptotic effect of MALP-2 may have affected microvascular angiogenesis by causing enzymatic separation of microvascular fragments and hypoxia in the initial phase after stent implantation. These additional stresses may have made the microvascular fragments more sensitive to the apoptotic effect of MALP-2, thereby detrimental to the angiogenesis of this implant (77). The questions of how to avoid the adverse impact of MALP-2, maximize the pro-angiogenic effects of MALP-2 and select a more appropriate implant material require further research and evaluation.

MALP-2 is a well-characterized TLR2/6 agonist with regenerative properties. On the one hand, TLR2 activation is necessary for endothelial cell migration. MALP-2 promotes the proliferation and migration of vascular endothelial cells through TLR2/6 and induces endothelial cells to release GM-CSF to promote angiogenesis. On the other hand, continuous TLR stimulation can aggravate inflammation, in which case blocking TLR2 may be a promising therapeutic intervention strategy (73, 78). Experimental loss-of-function studies in mice, mainly on TLR2 and TLR4, demonstrated that inhibition of TLR2 was beneficial in the pathology of atherosclerosis and ischemia/reperfusion injury after myocardial infarction, similar to the finding that long-term administration of TLR2 agonists significantly increased experimental atherosclerosis (79–82). Interestingly, short pretreatment with the TLR2 agonist Pam3 reduced chemokine CXCL1 release from cardiomyocytes, which limited subsequent leukocyte infiltration and could reduce myocardial infarct size and improve cardiac function after ischemia/reperfusion (83). Therefore, the administration or blocking of MALP-2 in a timely and controlled manner may positively affect the treatment of some cardiovascular diseases.

### 3.5 MALP-2 accelerates wound and bone healing

MALP-2 induced changes in the levels of peripheral blood cell populations in mice. A single intravenous injection of 10 μg MALP-2 resulted in a significant increase in F4/80+CD11b+ monocytes and Gr-1HighCD11b+ granulocytes, particularly in hematopoietic progenitor cells, which populations may promote wound healing in a paracrine manner (73, 84). Peptidoglycan is now commonly used to recruit and stimulate macrophages for accelerating wound healing, although peptidoglycan is difficult to purify and cannot be obtained synthetically. In contrast, MALP-2 is a synthesizable, highly purified substance with a well-defined receptor, a known inactivation mechanism by de-esterification and oxidation, and traceable *in situ* activity. Most MALP-2 activity is lost overnight in the wound, but approximately 25% is recovered from surrounding tissue after 24 hours, making MALP-2 even more advantageous in accelerating wound healing (85, 86).

MALP-2 stimulates the release of MCP-1 and other chemokines from skin fibroblasts and keratin-forming cells, which recruit granulocytes and monocytes/macrophages to the wound site. These cells continue to be activated by MALP-2 and release growth factors that accelerate wound healing. The protein array results indicated that MALP-2 also significantly promoted the secretion of G-CSF and IL-6 by endothelial cells, which mediated the improvement of endothelial healing (73, 87) (**Figure 3D**). MALP-2 inactivates leukocytes infiltrating the wound *in situ* by deacylation and oxidation of the thioether group, and MALP-2 injected into the wound acts as an indirect inducer of leukocytes to induce macrophages to synthesize the growth factors required for wound healing (86, 88). In summary, the mechanisms and processes of wound healing action are complex and interconnected.

Deiters U et al. used MALP-2 to improve the healing of full-thickness excision skin wounds in an obese diabetic mouse model. The results showed that the healing quality of the treated wounds was no different from that of the control group. However, the mice healed two weeks earlier and had approximately 25% residual MALP-2 activity after 24 h. These results suggest that MALP-2 can rapidly initiate the successive natural steps of wound healing and act as a healing facilitator in diabetic mice without side effects (87). Subsequently, Niebuhr M et al. conducted a phase I clinical trial of intradermal MALP-2 in 12 patients to study its tolerability when applied to human skin wounds, confirming that MALP-2 causes transient erythema and that patients can tolerate doses of up to 1 μg of MALP-2 without any systemic side effects. The local erythema induced by MALP-2 may be suggestive that wound healing is accelerating, as the erythema causes the recruitment and activation of macrophages and PMN cells, thereby inducing additional inflammatory responses. These cells increase the expression of growth factors in chronic wounds with lower levels of inflammation (88). These animal and human experiments confirm that MALP-2 does have a facilitative effect on wound healing, and MALP-2 is present only for hours, but its effects last for days. Routine human wound healing often does not require specific drugs. However, for many patients with chronic wounds that heal very slowly or not in clinical practice, such as diabetes, venous ulcers, and pressure ulcers, the application of MALP-2 offers a new therapeutic direction for these unique patients.

The healing effects of MALP-2 have also been explored in other human tissues. Bone healing *in vitro* studies showed that MALP-2 effectively induced bone resorption in mouse skulls by inducing the release of PGE and IL-6, increased the bone resorption activity of osteoclasts isolated from rat bone, and ultimately promoted bone healing (**Figure 3E**). However, MALP-2 could not increase the number of osteoclasts in bone marrow culture (89). Topical application of MALP-2 in mice revealed a decrease in biomechanical stiffness, a significant increase in HO-1 expression in the tendons, and a reduction in cyclin D expression, all of which delayed the healing of the injured tendons. It is likely that MALP-2 stimulated increased intracellular pressure and inhibited cell proliferation in the injured tendon, a naturally dystrophic tissue (90). Thus, MALP-2 is not applicable in the treatment of tendon injury, and perhaps MALP-2 has unexpected applications in more tissues, which will need to be explored later.

## 4 Shortcomings and prospects

MALP-2, a lipopeptide originally derived from *M. fermentans*, has been proven to activate a range of immune cells. MALP-2 indirectly enhances the cytotoxicity of NK cells to exert anti-tumor effects. MALP-2 activates PMN, which has anti-infective effects and improves survival in infected mice. MALP-2 stimulates endothelial cells and fibroblasts to release various cytokines and growth factors that shorten wound healing time and accelerate angiogenesis. MALP-2 matures dendritic cells and enhances antigen presentation to make them effective in vaccine adjuvants. MALP-2 has a promising clinical future with excellent results in animal experiments as a vaccine adjuvant and prophylactic agent for pulmonary infections. Its unique regenerative characteristics are closely related to its ability to recruit and stimulate macrophages to the wound, which are a significant source of several growth factors required for wound healing, and MALP-2 attracts and stimulates PMNs to assist in the healing process by preventing infection and wound debris removal. Thus MALP-2 has shown exciting accelerated effects in the animal experiment on wound healing, vascular healing, and bone healing, as well as in phase I clinical trials in human wounds.

However, there are limitations and contrasting aspects of MALP-2 studies. The dual role of MALP-2 as a TLR2/6 agonist that modulates the inflammatory response may exacerbate the inflammatory response and, thus, the disease progression. In terms of tumor suppression, there are different signaling types on the surface of the tumor, and even some tumors express TLRs on the surface. The development of tumors is intricate and complex, and the multiple cell types excited by MALP-2 also produce complex pleiotropic effects *in vivo*. Does MALP-2 have a remarkable inhibitory effect on every tumor? The safety of concomitant use with different chemotherapeutic agents, the optimal timing of MALP-2 stimulation, and the prognosis of MALP-2-treated tumors require plenty of substantial follow-up studies. MALP-2-treated tumors require a great deal of subsequent research. MALP-2 promotes wound healing and angiogenesis, but it is also unsuitable for all implant tissues, and its pro-apoptotic effects may adversely affect the implant’s stability *in vivo*. In conclusion, MALP-2 must further validate the specific function and safety in the human body.

TLRs 1, 2, 4, 5, and 6 recognize molecular patterns associated with bacterial pathogens, where recognition of microbial lipopeptides carrying only two fatty acids requires co-expression of TLR2 and TLR6. In addition to MALP-2, agonists of TLR2/6 include FSL-1 (Pam2CGDPKHPKSF), Pam2Cys, and Pam2CSK4 (Pam2), which share the same frame structure as MALP-2, but have peptides with different amino acid sequences and lengths (86). FSL-1 is a synthetic lipoprotein that represents the N-terminal part of the 44-kDa lipoprotein LP44 of Mycoplasma salivarium (91). FSL-1 stimulation induces a MyD88-dependent signaling cascade leading to AP-1 and NF-κB activation and the subsequent cytokine production (92, 93). The research found that FSL-1 enhances the phagocytosis of bacteria by macrophages and promotes dendritic cell maturation. It also has anti-tumor and pro-tumor activities and has applications in vaccine adjuvant and anti-HSV-2 infection (94–98). Pam2Cys and Pam2 stimulate splenocytes and macrophages more than Pam3, thus exerting adjuvant efficacy. Both Pam2Cys and Pam2 exert immune functions by binding TLR2/6 and have some roles in preventing respiratory infections, etc. However, their applications are narrower than those of MALP-2. Unlike MALP-2 and Pam2Cys, Pam2 has no cytokine-induced effect on dendritic (30, 99–102). The structural differences between the TLR2/6 agonists may explain their different directions of application in the future.

*M. fermentans* is susceptible to immunosuppressed patients and can easily exacerbate their disease, thus affecting the prognosis. Rheumatoid arthritis and AIDS are reported to be frequently associated with *M. fermentans* and its gene products (4). Future applications of MALP-2 in immunodeficiency models are a promising research point. MALP-2 also offers a new adjunctive treatment for patients with very slow or non-healing wounds in clinical practice. Studying the immune mechanisms and applications of MALP-2 in animals and humans will help to complete the mechanism of mycoplasma pathogenesis and facilitate the application of MALP-2.

## Author contributions

DL and XS drafted the manuscript and designed the figure. JW, JY and HL revised the manuscript. WT and ZY conceived the topic, JH was responsible for the supervision. All authors contributed to the review and approved the submitted manuscript.
